# The long-term impact of obesity in pregnancy on offspring hypothalamic feeding pathways

**DOI:** 10.1098/rsos.250681

**Published:** 2025-11-12

**Authors:** Pit Shan Chong, Laura Dearden

**Affiliations:** ^1^ Institute of Metabolic Science, University of Cambridge, Cambridge, UK

**Keywords:** obesity, pregnancy, hypothalamus, feeding, developmental programming

## Abstract

An accumulating body of evidence shows that offspring exposure to maternal obesity in the peri-natal period causes an increased risk to develop obesity later in life. Animal models have demonstrated that increased weight gain in offspring exposed to maternal obesity is preceded by increased food intake, implicating altered brain control of food intake as a likely cause. The hypothalamus is crucial for regulating feeding behaviour and energy homeostasis. This article reviews findings from human and animal studies to provide an updated perspective on how maternal obesity alters fetal hypothalamic development, predisposing offspring to long-term metabolic dysfunction. We discuss how maternal obesity impacts on hypothalamic development and the key molecular mechanisms, including epigenetic modifications, hormonal disruption, neuroinflammation and gut-brain axis interactions, which may mediate these changes. We highlight the critical gaps that remain in understanding the specific molecular pathways driving neurodevelopmental alterations in offspring, as well as emerging areas of research, including the role of extracellular vesicles in maternal–fetal communication. An in depth understanding of the molecular mechanisms that mediate the link between maternal metabolic state and offspring hypothalamic control of feeding is crucial in informing public health policies and clinical interventions aimed at reducing the intergenerational transmission of obesity.

## 1. Introduction

### 1.1. Multi-faceted causes of the global rise in obesity rates

In recent decades worldwide obesity levels have increased exponentially, leaving us in the midst of an obesity epidemic. Obesity is a major risk factor for a number of chronic diseases, including cardiovascular disease, diabetes and cancer and can also have a negative impact on mental health and wellbeing. Because of these associated health concerns, obesity represents a significant financial burden [1]. The financial and societal cost of obesity is particularly concerning for low and middle income countries that are starting to see rising obesity rates, in addition to countries like the UK and USA that have been experiencing rising obesity for decades.

There are many environmental factors driving increased obesity in the population. A changing food environment is a major driver of the increasing prevalence of obesity, as our modern industrialized food system allows increased availability of inexpensive and convenient ultra-processed foods [2]. Furthermore, socioeconomic status is strongly associated with obesity rates. Food insecurity (or food poverty) is associated with obesity among women in high-income countries [3]. Experiments on animals suggest that unpredictable food causes animals to store more energy in adipose tissue, and lower energy expenditure sufficient to fuel weight gain in the absence of increased food intake [4]. An individual’s body weight and metabolic health is a sum of their energy input (diet) and energy output (energy expenditure including physical activity). While some studies have suggested that a decline in physical activity levels in recent decades is to blame for rising body weight in the population [5], others have argued that there has not been a decline in energy expenditure [6], and it is instead a decline in basal expenditure in cells that has contributed to a reduction in daily energy expenditure [7].

However, not all individuals gain weight in the face of the ‘obesogenic’ environment that many of us live in. Obesity is therefore increasingly being recognized as a disease determined by risk factors linked to an individual’s biological make-up that interact with societal and environmental factors. Large genome-wide association studies have identified more than 200 common genetic variants linked to increased weight gain, primarily in genes related to brain function [8], which support the theory that obesity is caused primarily by changes in feeding behaviour that lead to excess food consumption and adipose tissue deposition. Although an individual’s genetics undoubtedly play a strong part in determining body weight, to date, the identified genetic changes can only account for small increases in body weight, and even when multiple genetic variants are combined into a genetic risk score, they explain a very small amount of the variance in Body Mass Index (BMI; [9]). There must therefore be additional mechanisms that can alter an individual’s biology—independent of their genetics—and increase the susceptibility to develop obesity and associated metabolic diseases. Indeed, it has recently been shown that the genetic heritability of child BMI is altered by an obesogenic home environment [10], demonstrating the intricacy and importance of gene and environment interactions in determining obesity rates.

Increasing evidence suggests that some of the variation in biology that underlies altered obesity risk comes from the environment experienced by an individual during their early life, roughly from conception to the age of 2 years. Changes to normal development during this time can influence long-term obesity risk (among other diseases), a concept embodied within the developmental origins of health and disease (DOHaD). An association between the fetal environment and later incidence of metabolic disease was first reported by Hales and Barker, who proposed the ‘thrifty phenotype hypothesis’ based on their observations of a link between reduced birth weight and increased cardio-metabolic disease in adulthood in a UK-based cohort of babies born in the 1950s [11,12]. Further studies examining individuals who were *in utero* during the Dutch Hunger Winter confirmed an association between *in utero* nutrition state and the development of metabolic disease, and suggested it was a causative relationship dependent on specific windows of exposure [13].

### 1.2. Rising obesity rates during pregnancy and associated health outcomes

Early studies in the DOHaD field investigated the long-term consequences of under-nutrition or intra-uterine growth restriction on long-term metabolic health. Over the past 20 years, numerous studies in human and animal models have explored the relationship between maternal obesity and/or gestational diabetes mellitus (GDM) in pregnancy and long-term health in offspring [14]. The global rise in obesity means there has inevitably been an increase in the number of pregnancies in women living with obesity. In the year 2024, obesity rates in women entering pregnancy were as high as 31% in areas of the UK, with a high percentage of the population living in deprived quintiles [15]. Women with type 2 diabetes now make up 56% of diabetic pregnancies (compared with 47% in 2014); they face additional healthcare inequalities, with 63% living in the two most deprived quintiles (compared with 7% in the least deprived quintile) and 53% belonging to ethnic minorities [16]. Thus, negative health outcomes associated with obesity and GDM in pregnancy are likely to impact most heavily on the most deprived people in society, and compound other socio-economic risk factors associated with obesity.

Obesity during pregnancy is associated with negative health outcomes for the mother and the baby both during pregnancy and birth. For the mother, obesity is associated with greater odds of developing GDM, which affects 10–20% of pregnancies in the UK and is itself associated with health risks for mother and baby [17]. Obesity in pregnancy also increases maternal hypertension and the risk of developing the life-threatening condition pre-eclampsia. For the fetus, maternal obesity is associated with an increased risk of stillbirth, being born both small or large for gestational age, and an increased incidence of shoulder dystocia and emergency caesarean birth [17]. As well as these immediate effects on the health of the mother and the baby, exposure to maternal obesity during pregnancy is associated with long-term health problems in offspring. There is now strong evidence that exposure to maternal obesity causes an increased risk to develop obesity, diabetes and cardiovascular disease later in life.

### 1.3. Increased obesity risk in children born from pregnancies complicated by obesity and gestational diabetes

One in three children leaving primary school in the UK are overweight or have obesity by the age of 11 [18]. This is concerning for the future health of the nation, as childhood obesity is a strong predictor of adult obesity and associated co-morbidities. Exposure to maternal obesity and/or GDM in pregnancy may be a contributing factor to rising childhood obesity rates. A recent meta-analysis of 67 birth cohorts from around the world calculated a 264% increase in odds of childhood obesity when mothers have obesity before conception [19]. While birth cohorts of this type also capture the effects of inherited BMI determining genetics and the shared household environment, studies in siblings discordant for exposure to maternal obesity or GDM have shown that there is an additional impact from the *in utero* environment [20,21].

### 1.4. Feeding behaviour and neuronal responses to food cues in offspring born from pregnancies complicated by obesity or gestational diabetes mellitus

Increased BMI in children born from pregnancies complicated by obesity or GDM could be driven by increased food intake. A recent study has shown increased food cue activation in the pre-frontal cortex of children of parents with obesity, which could underlie over-consumption of food. Offspring pre-frontal cortex activation shows a stronger association with maternal rather than paternal weight [22]. This suggests that there are additional factors resulting from maternal metabolic state that can influence brain responses to food in the offspring in addition to inherited genetics. A unique study of children born from pregnancies complicated by GDM demonstrated that GDM-exposed children have increased hypothalamic blood flow (a marker of hypothalamic activation) in response to glucose when compared with unexposed children, and that a greater hypothalamic response to glucose predicted greater increases in later BMI. These results remain significant after adjustments for child age, sex, current BMI and maternal pre-pregnancy BMI [23]. Fetal exposure to GDM is also associated with greater daily energy intake, and children exposed to GDM that was diagnosed early in pregnancy displayed greater food cue reactivity in their orbital–frontal cortex. Children exposed to GDM also had a larger waist-to-hip ratio, which was explained by their higher energy intake [22,24].

Unfortunately, there are few studies in humans examining whether altered feeding behaviour is a driver of obesity in individuals exposed to maternal obesity, owing to the difficulty in studying the direct consequences of obesity in pregnancy independently of genetics and household environment. Animal models in which genetic and environmental factors can be more tightly controlled have shown conclusively that maternal obesity in pregnancy, with or without GDM, results in offspring hyperphagia and that this is a driver of increased adiposity. This altered feeding behaviour is evident from a young age; rodent offspring of obese dams consume more milk during experimental tests of independent feeding, even after correcting for their body weight [25]. In rodents, independent feeding by pups (i.e. calorie intake other from dam milk) typically emerges during the third postnatal week. The offspring of obese dams transition earlier to independent feeding and although they consume the same amounts of milk from the mother, they supplement milk intake with additional solid food consumption, suggesting impaired satiety signals [26]. Animal models have shown that not only does maternal obesity during pregnancy programme hyperphagia, but also increases preference for high-fat foods in non-human primates (NHP) [27], rats [28] and mice [29].

### 1.5. Importance of the hypothalamus and correct hypothalamic development in maintaining energy homeostasis

Animal models that show increased weight gain in offspring exposed to maternal obesity are preceded by increased food intake, implicating altered neuronal control of food intake as an underlying cause. The hypothalamus is the primary site in the brain for maintaining energy homeostasis, which it does by appropriately adjusting parameters—including food intake-based on signals of nutrition state received from the periphery. The primary neuronal populations in the hypothalamus involved in food intake control are orexigenic neurons expressing neuropeptide-Y (NPY) and/or agouti-related peptide (AgRP), and anorexigenic neurons expressing pro-opiomelanocortin (POMC). Both of these populations of neurons are resident in the arcuate nucleus of the hypothalamus (ARC) adjacent to the median eminence (MEE) where the blood-brain barrier (BBB) is leaky, allowing cells to sense peripheral circulating nutrients (e.g. glucose, fatty acids) and hormones (e.g. leptin, insulin, ghrelin). Interestingly, these neuronal populations that are well known to control opposing actions for food intake after birth, initially form from a common lineage [30] and therefore, developmental disruption around the time point of switch from a POMC-defined neuron to an NPY-defined neuron (E13 in mice) is a window for potential disruption. In rodents, melanocortin neurons are born between days E11–12 [30,31] and the circuitry of projections from these neurons is completed in the post-natal period [32]. In humans and NHP, which give birth to offspring with more mature brains, the majority of the circuit formation is completed by birth. However, there is still evidence of refinement of the melanocortin circuitry in NHP in the early post-natal period [33].

AgRP/NPY and POMC neurons project intra-hypothalamically to regions, including the para-ventricular nucleus of the hypothalamus (PVH) where they synapse onto melanocortin 4 receptor expressing neurons. It is well-established in rodents and humans that disruption of signalling within these key pathways in the hypothalamus results in obesity [34]. More recent studies have shown that genetic disruption of the cell signalling pathways required for correct hypothalamic development specifically axon guidance pathways—result in severe early onset obesity in humans [35] and impaired glucose tolerance in rodents [36]. Therefore, changes to hypothalamic development caused by an altered *in utero* milieu of a pregnancy complicated by obesity could be a contributing cause to rising obesity rates via life-long changes in hypothalamic function.

## 2. Impact of maternal obesity on development of the hypothalamus

Changes to offspring hypothalamic development in the perinatal period as a result of altered nutrition and/or hormonal signalling in response to maternal obesity is a likely cause of long-term metabolic phenotypes in offspring. Examining molecular and detailed anatomical changes in the hypothalamus requires access to brain tissue and animal models must be used for this purpose. Murine hypothalamic development is well defined, allowing researchers to distinguish changes from the normal process of neurodevelopment. Therefore, the following section refers primarily to studies examining the impact of maternal obesity on hypothalamic development, mainly in rodents.

### 2.1. Neurogenesis and cell fate decisions

The neuroendocrine portion of the hypothalamus—which is the primary site of feeding regulatory pathways—comprises the anterior and tuberal hypothalamus. In the mouse tuberal hypothalamus, all neurons are produced during a period of only a few days [37]. Experiments using 5-bromo-2′-deoxyuridine (BrdU) labelling of newborn cells have shown that the majority of neurons in the PVH and dorsomedial nucleus (DMH) are generated between E12–E14, whereas the ARC and ventromedial nucleus (VMH) have longer periods of neuronal generation from E12–E16 [30,31].

#### 2.1.1. Decreased proliferation of neural progenitor cells in the hypothalamus

Maternal obesity causes a reduction in the proliferative potential of hypothalamic neural progenitor cells generated from the fetal hypothalamus and decreased neurogenic markers in newborn mice in the mid-gestation period [38,39]. Although this period is when the majority of hypothalamic cells are born, hypothalamic cell turnover and neurogenesis continue in the post-natal period and into adulthood in mice [40]. A recent study found that in male offspring of high-fat diet (HFD)-fed mothers, newly born cells (as labelled by BrdU) were significantly decreased in the ARC, but not other hypothalamic regions, such as the VMH or DMH, on postnatal day (PND) 9. However, by PND21 decreased proliferation was observed in all hypothalamic regions of both male and female offspring exposed to a maternal HFD [41]. These results suggest there are different time windows of vulnerability within the hypothalamus, with the ARC being sensitive at an earlier time point than other regions of the hypothalamus. Furthermore, it also suggests time differences in windows of sensitivity between male and female offspring. This study also reported upregulation of pathways related to neuronal death in offspring, suggesting that maternal nutrition may interfere with cell survival as well as proliferation [41]. This is consistent with previous reports that HFD induces apoptosis of hypothalamic neurons in rodents [42].

#### 2.1.2. Changes in pathways that regulate differentiation of neural progenitor cells

The Notch signalling pathway is a key regulator of neurogenesis in the central nervous system. As Notch signalling inhibits pro-neural genes, models lacking Notch signalling exhibit an increase in neurons throughout the embryo [43]. Mice which lack Notch signalling in the hypothalamus demonstrate that Notch signalling is essential for the differentiation of late ARC neurons in the mouse from E13 [44]. Exposure to maternal obesity results in an upregulation of Notch signalling in the neonatal period [38], and a concomitant decrease in expression of the pro-neural transcription factor Mash1. As the Notch/Mash1 pathway contributes to neuronal differentiation into POMC and NPY lineages [44,45], these signalling disruptions could therefore be an underlying cause of altered anorexigenic/orexigenic cell populations in the offspring of obese mothers. Rodent models of obese pregnancies have consistently shown that offspring of obese or HFD-fed mothers have a higher ratio of AgRP/NPY to POMC-expressing neurons [38,46], which may be caused by a reduction in the differentiation of newborn neurons into POMC neurons [41].

Changes in proliferation of neural stem cell populations and downstream differentiation pathways may also result in long-term changes to cell types in the hypothalamus. Astrocytes, once viewed merely as support cells, are now recognized as active modulators of neuronal function and synaptic plasticity. Several studies in rodents have shown a decrease in cells adopting a neuronal fate, and concomitant increase in the proportion of newly born astrocytes in the developing fetal and neonatal mouse hypothalamus caused by maternal obesity in pregnancy [47]. This abnormal increase in astrocyte numbers may disrupt the proper assembly and connectivity of hypothalamic circuits, which are critical for regulating feeding behaviour and energy homeostasis. Interestingly, recent single-cell sequencing analysis in offspring exposed to maternal obesity only in the post-natal period showed no difference in relative cell populations [48], probably owing to the fact that neurogenesis and differentiation are mostly completed by the post-natal period. This again highlights specific windows of vulnerability for different components of the hypothalamic machinery that controls energy homeostasis.

### 2.2. Hypothalamic circuitry and anatomical development

The hypothalamus is a highly connected region of the brain. Animal models of maternal obesity have shown numerous changes to the wiring of the hypothalamus, primarily focused on intra-hypothalamic connections but also in connections to other parts of the brain. Experiments using neonatal hypothalamic explants demonstrate that offspring exposed to maternal obesity or HFD feeding show reduced neurite extension *in vitro* [49]. Reduced neurite outgrowth may be caused by altered expression or responsiveness to classical axon guidance cues. During normal development, neurites from NPY neurons in the ARC are repelled away from the ARC, in part owing to Netrin-1 expression, to head towards their target areas, such as the PVH. In fetuses developing in obese mothers, this repulsive force may not occur because of the overexpression of *Dcc*, which mediates attraction and reduced expression of *Unc5d*, which mediates repulsion. Consequently, NPY neurites may have a reduced ability to leave the ARC, which reduces their ability to reach the PVH [50]. Changes in the density of intra-hypothalamic projections may also be related to altered sensitivity to hormones such as leptin, as described below (§3.2). Consistent with reduced neurite extension, neural projections from the ARC to the PVH are markedly reduced in the offspring of diabetic [51], obese [38] or HFD-fed mothers [46]. Furthermore, translatome analysis of ARC POMC neurons in offspring after maternal HFD exposure reveals significantly impaired spatial distribution and axonal extension, without altering POMC cell number in the ARC [52].

Changes in feeding-related neurocircuitry in offspring exposed to maternal obesity are not limited to projections between the ARC and PVH. A rat model has demonstrated increased intra-hypothalamic input to the lateral hypothalamus (LHA) in exposed offspring that was associated with changes in leptin sensitivity [53], and a separate study in mice showed LHA neurons receiving synaptic input from the bed nucleus of the stria terminalis have enhanced excitatory input following exposure to maternal overnutrition. This persisting enhancement of excitatory drive within LHA circuitry could be an underlying cause of increased responsiveness to food cues in offspring of obese mothers [54].

A recent mouse study has demonstrated a previously unknown role for microglia in shaping hypothalamic development. Microglia transiently accumulate in the mediobasal hypothalamus during neonatal life, playing a key role in synaptic pruning and the refinement of perineuronal nets (PNNs) [55]. Maternal HFD during lactation enhances microglial activation, leading to prolonged synaptic engulfment and PNN overabundance, which impairs the establishment of brain-to-pancreas circuits critical for insulin secretion in adulthood, whereas depleting microglia during PND6-16 results in an overabundance of PNNs and impaired glucose tolerance in adulthood [55]. This shows that microglial sculpting of neuronal circuits during early life is a previously unappreciated critical regulator of metabolic programming, which can be impacted by maternal metabolic state.

In humans, it is not possible to visualize precise connections between different parts of the hypothalamus in living subjects. However, changes in AgRP projections between the ARC and PVH have been observed in offspring in a NHP model of maternal obesity [56]. In humans, the only current data on changes in hypothalamic circuitry or anatomy comes from functional magnetic resonance imaging analysis of hypothalamic microstructure. Mean diffusivity (MD) describes the amount of water diffusion within brain tissue and can be used to examine differences in brain structural integrity. In humans, hypothalamic MD in newborns is positively correlated with later childhood adiposity. A prospective longitudinal study in two independent cohorts of mother–infant pairs observed a positive linear association between maternal pre-pregnancy BMI and newborn offspring hypothalamic MD [57]. While the prospective study design cannot formally establish causality, it may help disentangle the temporal sequence of effects, as offspring hypothalamic MD was characterized shortly after birth, before significant exposure to the postnatal environment. Maternal circulating free fatty acid levels during pregnancy are also associated with newborn hypothalamic MD [58], demonstrating that maternal metabolic factors in addition to BMI can alter offspring hypothalamus.

### 2.3. Blood-brain barrier disruption

In adult mice fed an HFD, one of the hypothalamic abnormalities that is thought to contribute to worsening energy homeostasis is damage to the BBB at the MEE–ARC interface. BBB permeability in the ARC is determined by capillary endothelial cells and tanycytes. Tight junctions between endothelial cells limit paracellular entry of blood-borne molecules into the brain, whereas endothelial cell transporters and fenestrations regulate transcellular entry. Tanycytes form a further barrier that prevents free diffusion of blood-borne molecules. BBB permeability is increased in neonatal offspring of obese dams, as assessed by Evans Blue diffusion into the ARC [59], possibly owing to a reduced number of tanycytic processes in the ARC of offspring after exposure to maternal obesity. Interestingly, rodent models suggest that maternal obesity does not alter the total number of blood vessels irrigating the ARC but rather increases the proportion of permeable vessels [59]. Obesity and diabetes during pregnancy are both associated with transient neonatal hypoglycaemia at birth, and dips in blood glucose levels have been suggested to trigger the release by tanycytes of vascular endothelial growth factor (VEGF)-A, the main growth factor known to promote endothelial cell fenestration [60]. Therefore increased tanycytic release of VEGF could explain the changes in BBB permeability seen after birth in offspring from pregnancies complicated by maternal obesity or diabetes. A further cause of disruption to the offspring BBB could be increased circulating fatty acids, which have been shown to promote functional and structural changes in the MEE [61]. The consequences of disrupted BBB integrity are long-lasting. Not only might disrupted BBB contribute to the development of resistance to circulating hormone signals such as leptin and insulin (discussed further below), altered integrity of BBB is also associated with long-term metabolic changes. Interestingly, cross-fostering of neonatal offspring of obese mice to a lean dam is able to rescue both the disruption of BBB and some accompanying metabolic phenotypes [62], demonstrating that any programmed damage is reversible.

## 3. Mechanisms mediating the effects of maternal obesity on offspring hypothalamus

Changes to the normal metabolic milieu of pregnancy can influence offspring hypothalamic development, modulating hypothalamic function and elevating the risk of metabolic disorders in children. In order to stop the inter-generational transmission of obesity risk, researchers must first identify the molecular mechanisms mediating disrupted hypothalamic development and thus changes to long-term hypothalamic function. This section will discuss the potential underlying mechanisms affecting hypothalamic development in offspring owing to maternal obesity.

### 3.1. Epigenetic modifications

Fetal development is a critical window for epigenetic programming. Epigenetic modifications such as DNA methylation, histone modifications, and the activity of small non-coding RNAs have been proposed as a mechanism linking maternal obesity and offspring development, and as a critical mechanism for early neurodevelopmental reprogramming [63]. The correction of maternal obesity pre-pregnancy by bariatric surgery results in a change in methylation status of key genes required for glucose homeostasis in offspring [64]. Furthermore, evidence is emerging that epigenetic marks can persist over multiple generations [65], thus contributing to trans-generational transmission of obesity risk.

Evidence from both human and animal studies suggests the *Pomc* gene is particularly susceptible to epigenetic regulation in the peri-natal period [66,67]. Exposure to an HFD during gestation can induce permanent hypermethylation in the *Pomc* promoter and enhancer regions, resulting in altered integration of leptin signalling in the hypothalamus [68] and suppression of satiety response [69]. These epigenetic alterations are associated with weight gain, increased adiposity and insulin resistance. Changes in DNA methylation may also be a source of sex differences in the offspring phenotype in response to maternal obesity [70]. A recent study in rats demonstrated that hypothalamic insulin receptor expression and DNA promoter methylation are sex-specifically altered in adult offspring of HFD-fed mothers, with males showing more pronounced changes in methylation that are associated with insulin resistance and metabolic dysfunction [71].

Micro-RNAs (miRNAs) are small non-coding RNAs that post-transcriptionally modify target gene expression. miRNAs are a promising candidate for translating dynamic changes in nutritional state into changes in genomic regulation in offspring. A recent human study has shown significant differences in the expression of candidate miRNAs in circulating blood samples of newborn offspring dependent on maternal BMI [72] or GDM [73]. Studies in animals have begun to explore and demonstrate causative roles for altered miRNA expression in mediating some of the effects of maternal obesity on offspring phenotype. Exposure to maternal obesity causes upregulation of miR-505-5p in the fetal hypothalamus, and downregulation of miR-505-5p targets that are related to fatty acid sensing pathways [29]. Over-expression of miR-505-5p in the adult mouse brain causes increased intake of an high-fat food pellet, suggesting that programmed over-expression of this miRNA resulting in altered hypothalamic fatty acid sensing could underlie altered food choices in offspring that lead to obesity. Interestingly, increased expression of miR-505-5p is rescued in a model of maternal exercise that improves insulin sensitivity without weight loss [29], suggesting that targeting maternal and/or fetal insulin levels is a potential intervention strategy to mitigate the long-term health risks faced by offspring.

### 3.2. Hormonal disruptions: insulin, leptin and ghrelin

Pregnancy is a time of high energy demand for the mother, and as a result the body makes a series of metabolic adjustments in pregnancy to support the growing fetus. Many metabolic hormones are altered in obesity and thus remain altered- or are further dysregulated—in a pregnancy complicated by obesity or GDM. Some of these key metabolic hormones have dual roles in coordinating neurodevelopment of the hypothalamus. Therefore, changes in hormone levels in the mother and fetus can impact hypothalamic development and result in permanent metabolic dysfunction in offspring.

#### 3.2.1. Leptin

Leptin is a key anorexigenic hormone that plays an integral role in energy balance by acting on the hypothalamus to modulate food intake and energy expenditure. Leptin signalling suppresses NPY/AgRP neurons that stimulate hunger, activates POMC neurons that drive satiety and provides a negative feedback system that balances energy intake and expenditure. Beyond energy homeostasis, leptin is critical for neuronal development, particularly in circuitry related to hypothalamic regulation of feeding behaviour. The formation of projections from the ARC to the PVH during development is a critical process regulated by leptin. In rodents, a post-natal increase in leptin levels is required for the proper development of ARC-PVH projections [32]. Modulation of offspring post-natal leptin levels by maternal obesity and/or postnatal overnutrition can impact the proper wiring of the feeding circuits [74,75]. Interestingly, in leptin-deficient (Lep^ob/ob^) mice, exogenous leptin treatment can restore these projections when administered only during a short window of the postnatal developmental period, emphasizing the requirement of temporal specificity for leptin action on proper hypothalamic connectivity [76]. This crucial window for establishing ARC-PVH projections is defined by the formation of perineuronal nets [77]. However, whether the formation of perineuronal nets is altered with exposure to maternal obesity remains to be investigated. Molecular studies have shown that leptin promotes neurite formation by promoting the formation of axonal growth cones in neurons [78]. Leptin regulates the development of feeding circuits in the hypothalamus through a specific leptin receptor long isoform (LepRb), with distinct signalling pathways downstream of the receptor guiding the formation of NPY and POMC projections to different PVH neuronal compartments [79]. The involvement of leptin in establishing intra-hypothalamic circuitry is a probable source of nutrition-mediated disruption to these projections, particularly as maternal obesity leads to hypothalamic leptin resistance in postnatal offspring [49]. Leptin is also responsible for the formation of external feeding circuits into the hypothalamus, such as the Glucagon-Like Peptide 1 (GLP-1) receptor positive neurons originating in the Nucleus of the Solitary Tract (NTS) that innervate the PVH [80], but whether these extra-hypothalamic circuits are regulated in response to fetal nutrition are unknown.

#### 3.2.2. Insulin

Insulin, like leptin, exhibits both metabolic and neuromodulatory functions. Insulin is produced by the pancreas and in the fetal brain and is vital for neuroplasticity; variations in insulin sensitivity owing to age or metabolic conditions result in changes to neuronal structure and function. Insulin plays a crucial role in neuronal differentiation and synapse formation during fetal development. Early research demonstrated that insulin stimulates neurite outgrowth in cultured neurons [81,82]. Insulin receptor activation triggers downstream signalling pathways, including the Phosphoinositide 3-kinase (PI3K)- Akt and Mitogen Activated Protein Kinase (MAPK) pathways, which affect neuronal growth, survival and energy balance [83]. It is therefore significant that human fetuses from pregnancies complicated by maternal GDM or obesity exhibit whole body insulin resistance *in utero* [84]. Rodent models have shown that maternal obesity causes insulin resistance specifically in the fetal hypothalamus. This may be an underlying cause of structural and functional alteration in the hypothalamus [38,85], as well as in other circuits, such as the dopaminergic system, which regulates energy and glucose homeostasis [86]. High insulin levels in the offspring hypothalamus do not only cause problems in the fetal brain, persistent hypothalamic hyperinsulinaemia during the neonatal period results in lasting malformations in hypothalamic nuclei, which are associated with alterations in insulin receptor signalling and glucose homeostasis [87]. A study by Vogt *et al.* demonstrated that genetic knock-out of the insulin receptor on POMC neurons rescues the reduced projections of these neurons to the PVH in offspring exposed to maternal over-nutrition, suggesting that insulin signalling in the neonatal period is at least in part responsible for reduced POMC connectivity between the ARC and PVH, and associated dysglycaemia [46].

#### 3.2.3. Ghrelin

Ghrelin, secreted from the stomach, is well characterized as a stimulant of appetite and feeding behaviour through its action at growth hormone secretagogue receptors in the ARC. In normal pregnancy, ghrelin levels typically peak during mid-gestation and then decline [88,89]. However, in cases of maternal obesity, these levels may be lower or display varying patterns [90]. Previous studies have shown that maternal ghrelin is closely linked to fetal birthweight [91].

Research has indicated that ghrelin is required to restrict the formation of projections from the ARC to the PVH, and therefore loss of ghrelin signalling during the post-natal period in rodents results in increased density of projections between the ARC and PVH and associated metabolic dysfunction [92]. Furthermore, the study also showed that direct exposure of postnatal ARC neuronal explants to ghrelin inhibited axonal growth and obstructed the neurotrophic effect of leptin. Interestingly, the effects of neonatal ghrelin on body weight regulation seem to be sex-dependent, as treatment with ghrelin in female rats and anti-ghrelin in female mice does not lead to weight changes. Postnatal ghrelin resistance—characterized by reduced c-fos activation in NPY/AgRP neurons following ghrelin administration, has been associated with maternal obesity and postnatal overnutrition [93]. Given that ghrelin signalling is implicated in the neonatal programming of metabolism, these alterations in the ghrelin system may disrupt the normal regulation of feeding and energy balance in mice subjected to postnatal overnutrition.

### 3.3. Endoplasmic reticulum stress and unfolded protein response

Metabolic and nutritional factors, such as elevated glucose and lipid levels, can lead to endoplasmic reticulum (ER) stress [94,95]. Recent studies have demonstrated that maternal obesity induces hypothalamic ER stress in offspring [96] and that this is causally associated with disrupted energy homeostasis [49]. Studies using hypothalamic explants show that saturated fatty acids such as palmitic, lauric and myristic acids—which are increased in high fat diets and in animals consuming them—are a key driver of ER stress in neonatal hypothalamic cells. The observation that administration of the ER stress-relieving drug tauroursodeoxycholic acid rescues the reduction of neurite formation caused by saturated fatty acids [42], suggests that fatty acid-induced ER stress is a key mechanism by which perinatal nutrition impacts on hypothalamic circuitry.

Maternal obesity also disrupts the interaction between two vital stress response pathways: the unfolded protein response (UPR) and the heat shock response (HSR) [97]. The UPR is activated by the buildup of misfolded proteins in the ER, where it helps regulate protein homeostasis by enhancing folding capacity and limiting the synthesis of new proteins. Conversely, the HSR serves to protect cells from heat-induced protein denaturation and has a role in numerous neuronal cellular functions, including acting as a mediator in neuronal axon guidance [98]. Offspring exposed to maternal obesity have a disruption of these protective cellular responses, resulting in chronic hypothalamic ER stress, which is associated with altered intra-hypothalamic projections and hyperphagia [97]. Interestingly, research has shown that administration of 4-phenyl butyrate, a protein-folding chemical chaperone and UPR activator from PND4, can rescue metabolic outcomes in the offspring of obese mothers [96]. This suggests that modulating either ER stress or the UPR in offspring in the post-natal period could mitigate some of the negative effects of maternal obesity.

### 3.4. Neuroinflammation

ER stress is a key factor in triggering neuroinflammation, a process that can exacerbate the effects of maternal obesity on the offspring’s hypothalamus. The activation of stress pathways, including NF-κB and JNK, during ER stress, can promote glial cells to release inflammatory mediators such as interleukin-6 (IL-6 [99]) and IKKβ/NF-κB in the hypothalamus, which may be a driver of central insulin and leptin resistance [49,100,101]. Many studies have shown that higher maternal BMI is linked to increased placental production of pro-inflammatory cytokines, such as IL-6 and tumour necrosis factor-α (TNF-α) [102]. These cytokines may cross the placental barrier and access the developing fetal brain, activating glial cells, including microglia and astrocytes [103,104]. This activation initiates a cascade of inflammatory responses that may impair neuronal maturation, cause neuronal damage, and modify neural circuitry [102,105]. Gliosis refers to the brain’s response to local inflammation, which is characterized by increased proliferation of glial cells. Studies in both humans and animals suggest that hypothalamic gliosis is linked to obesity and related metabolic dysfunction. Children exposed to GDM *in utero* display evidence of hypothalamic gliosis after adjusting for child’s age, sex, BMI z-score and maternal pre-pregnancy BMI [101,106]. In these studies the evidence of gliosis was limited to the mediobasal hypothalamus, highlighting the vulnerability of the developing hypothalamus, and independent of current child adiposity.

In an NHP model of maternal obesity, an inflammatory milieu has been shown to disrupt the development of hypothalamic melanocortin circuits involved in appetite and energy homeostasis, increasing the offspring’s susceptibility to obesity, insulin resistance and other metabolic diseases [104]. Pro-inflammatory cytokines, such as IL-6, have been shown to play a critical role in hypothalamic development. Sanders *et al*. [50] demonstrated that maternal obesity results in increased IL-6 levels, which can deregulate gene expression in the ARC of the hypothalamus, as well as disrupt intra-hypothalamic connections to other hypothalamic regions [50]. Furthermore, IL-6 regulates neurite outgrowth, affecting the formation of normal neuronal connectivity in the hypothalamus, which is essential for the proper functioning of feeding circuits.

Dietary factors further exacerbate the impact of maternal obesity on hypothalamic development and inflammation. Pimentel *et al*. [107] revealed that maternal consumption of trans fatty acids during pregnancy and lactation stimulates hypothalamic inflammation in offspring through the TLR4/NFκB/p65 signalling pathway [107]. Trans fatty acids, commonly found in industrial food products, activate TLR4 receptors in the hypothalamus, triggering an inflammatory cascade that activates microglia and astrocytes. This inflammatory stimulus alters the development of hypothalamic circuits responsible for feeding behaviour, compromising the offspring’s ability to regulate food intake and energy expenditure. These findings underscore the importance of not only maternal weight, but also maternal diet as a key regulator of the inflammatory environment surrounding the developing brain and its long-term metabolic consequences. However, from current studies, it is not yet clear how much neuroinflammation occurs as a direct consequence of maternal factors on the offspring developing hypothalamus, and how much is secondary to other metabolic changes in the offspring.

### 3.5. Gut microbiota and the gut-brain axis

The gut microbiota, a significant player in the gut-brain axis, is increasingly being recognized as a potential mechanism through which maternal obesity can impact on offspring long-term metabolic control. Recent papers have thus addressed the consequences of changes to the microbiome on the offspring brain. A recent study in rodents demonstrated that microbiota alterations are correlated with brain metabolites, particularly those associated with metabolism commonly found in the hypothalamus [108]. Even short-term changes in maternal diet-induced microbiota are linked to metabolic alterations and behavioural disturbances observed in adolescent offspring, suggesting a potential interaction between microbiota and the hypothalamus that leads to enduring changes associated with feeding behaviour and metabolism [108].

Maternal consumption of low-calorie sweeteners (LCS) also leads to gut microbiota alterations that affect hypothalamic melanocortin circuits in offspring [109]. Phenylacetylglycine, a gut microbial co-metabolite, was identified as a mediator of these effects. Maternal LCS intake resulted in a rewiring of the hypothalamic melanocortin system, characterized by reduced POMC fibre density and increased AgRP fibre density in male offspring. These alterations were associated with increased adiposity, glucose intolerance and disrupted parasympathetic innervation of pancreatic β-cells in adult male offspring. Interestingly, these alterations were not observed in female offspring, indicating sex-specific vulnerabilities to changes in gut microbiota.

Considering the potential significance of maternal microbiota in influencing fetal brain development, the specific category of gut bacteria that impacts brain metabolism necessitates further investigation. Administration of the maternal microbiota *Bifidobacterium breve* to germ-free mice resulted in alterations to fetal brain metabolism and alleviated the detrimental effects of maternal obesity [110]. It remains unclear whether this occurs via a direct effect of microbiota supplementation on the fetal brain or via a change to maternal physiology. However, this finding highlighted the importance of recognizing potential protective bacteria within the gut that contribute to the preservation of fetal brain function in the context of maternal obesity.

## 4. Conclusion and future directions

**Figure 1.**
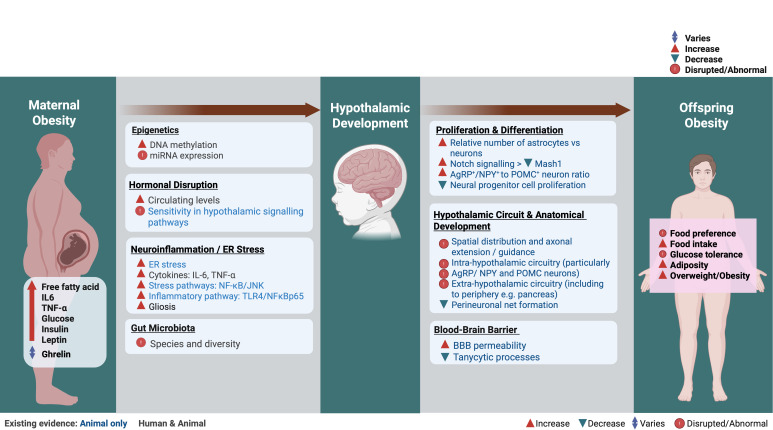
Obesity in pregnancy is associated with increased circulating levels of nutrients, hormones and inflammatory markers in the mother. Some of these directly pass to the fetus and can cause a variety of changes in the developing offspring hypothalamus, including influencing epigenetic marks, altering the sensitivity of hormonal signalling pathways and causing neuro-inflammation. Changes to these molecular mechanisms are associated with altered development of the offspring hypothalamus and subsequently altered feeding behaviour. While the link between exposure to maternal obesity and offspring obesity has been demonstrated in offspring, the majority of studies examining the molecular mechanisms underpinning this link have taken place in rodent models. We have highlighted where there is evidence of these processes in both humans and animal models. Figure created with Biorender.

Strong evidence suggests that maternal obesity both with and without GDM programmes lasting changes in metabolic regulation in offspring. As shown in figure 1 (left panel), the data available from human and animal studies show that maternal obesity in pregnancy is associated with epigenetic modifications, altered hormonal signalling and ER stress/neuroinflammation and that these factors together cause changes to the development of hypothalamic feeding pathways (figure 1, right panel) which drive later obesity. The studies presented here together highlight the critical influence of the intrauterine environment on lifelong health outcomes.

Despite significant advancements in understanding these mechanisms in the last decade, key questions remain unanswered. The extent to which these effects can be reversed through interventions targeting the mother or offspring is still unclear, as is the timing that any interventions would need to occur (pre-pregnancy, during pregnancy or in offspring after birth). While emerging research suggests that dietary modifications, physical activity and microbiome-targeted therapies may mitigate some of these adverse effects, further studies are needed to determine their efficacy, timing and long-term impact. The DOHaD field is only recently beginning to address sex-specific differences in susceptibility to metabolic programming [70] and further research into the hormonal and genetic influences that may shape differential responses to maternal obesity will be critical in developing effective intervention strategies.

While many studies have focused on feeding behaviour and the projection of AgRP/NPY and POMC neurons from the ARC to the PVH, relatively few studies have examined the effect of the peri-natal environment on other hypothalamic areas or other energy homeostatic processes, e.g. the circuitry required for hypothalamic glucose sensing. Advanced techniques like single-cell RNA sequencing will help to identify in an unbiased way the non-traditional hypothalamic cell types that are altered by changes in perinatal nutrition. Although we know astrocytes are impacted in addition to neurons (§2.1.2), limited studies have examined the impact of the peri-natal environment on other glial cell types within the fetal hypothalamus such as oligodendrocytes or tanycytes. Maternal HFD has been shown to reduce both immature and mature oligodendrocytes in the prefrontal cortex in adult offspring [111], but the effects on hypothalamic oligodendrocytes are yet to be examined. The shared developmental origin of neuronal cell types and the emerging essential role of glial cells in maintaining energy homeostasis makes them an important target for future research.

Another critical area for future research is the role of extracellular vesicles (EVs) in mediating maternal–fetal crosstalk. EVs, which carry bioactive molecules such as proteins, lipids and miRNAs, have emerged as key mediators of intercellular communication. During pregnancy, the placenta is a source of EVs that have been proposed to act on both the maternal [112] and fetal [113] brain. Given the recent report that EVs contain neural components such as potential trophic factors that could have a role in neurodevelopment [114], understanding whether the mechanisms of EV release and cargo of the vesicles are altered in the context of maternal obesity should be a key priority of future research and could reveal both new biomarkers for predicting offspring metabolic risk and inform interventions to block harmful signals.

The potential for trans-generational transmission of metabolic risk through epigenetic inheritance also warrants further exploration. Identifying whether these epigenetic marks can persist across generations—independent of associated phenotypes and gametes that are exposed owing to their development *in utero*—and whether targeted interventions in early life can prevent their transmission will be crucial for designing effective prevention strategies.

In conclusion, improving the health of mothers before and during pregnancy is essential not only for improving obstetric outcomes, but also for breaking the intergenerational cycle of obesity and metabolic disorders that contribute to the trapping of families within unhealthy lifestyles. A multidisciplinary approach, integrating insights from neuroscience, endocrinology, and public health, will be vital in developing targeted interventions that optimize fetal hypothalamic development and therefore long-term metabolic health. By bridging the gaps in knowledge and translating mechanistic research from animal models into clinical and public health strategies, we can work towards improving health trajectories for future generations.

## Data Availability

This article has no additional data.
